# Pediatrics a Specialty

**Published:** 1912-03

**Authors:** Clarence A. Rhodes

**Affiliations:** Atlanta, Ga.; Late Resident Physician I. N. Y. Foundling Hospital, New York City


### PEDIATRICS A SPECIALTY.

By Clarence A. Rhodes, A. B., M. D., Atlanta, Ga.
*Late Resident Physician I. N. Y. Foundling Hospital, New York City.*

Until recent years, no branch of medicine had been so neglected by the general practitioners, our medical schools, and Universities as that of pediatrics. In fact, to-day the older practitioners admit they know very little about the diseases of infancy and childhood, since these subjects were entirely neglected, or only superficially taught, during their medical course. However, to-day, things have changed, and more and more attention is given to what is now considered one of the most important branches in medicine, that dealing with the infant from the time of its birth until it reaches maturity.

Most practitioners claim, and even some pediatricians admit, that pediatrics is not a specialty, but a department of general medicine, but we support the contention that in the highest sense pediatrics is a specialty. True, not in the sense that ophthalmology and otology are regarded as specialties, because they have reference to special organs of the body, but in the broader sense, that pediatrics deals with all the organs of the body, but limited to the periods of infancy and childhood. A special branch of the general science of human life devoted to the interests of a special class, the most helpless and appealing of all, and it includes many kinds of inquiry into the normal and abnormal child. For the most part diseases occur in all periods of life, but with manifestations vastly different in the child than in the adult.

It must be conceded that nutrition, dietetics, and treatment in infancy and childhood is peculiarly a special study, inseparably united with the broad subjects comprised under the heads of hygiene, sanitation, social condition, economic, humanitarian, philanthropic, and individualistic, each of these factors must be realized and reckoned with.

The physician who would effectively and honestly practice in this field must, therefore, become a medical sciologist and economist. Economic and social problems should claim a large share of his attention; it is his duty to gather data for such investigations. Of what value are statistics of factors, or combinations of factors regarding pediatrics when compiled by those whose observation has been limited to the sick children, while investigation of environments have been wholly neglected or of only a superficial nature? Of what value are deductions from such statistics to uplift humanity, which should be the guiding star of all medical investigation? In addition, a successful practitioner of pediatric medicine should have a thorough knowedge of dairy chemistry, and of animal and dairy hygiene, because from this source is derived the sole food supply for a large percentage of those intrusted to his care. A technical knowledge of these subjects is fundamentally necessary for the intelligent modification of milk, without this knowledge success can not be assured. Every practitioner of medicine faces the milk problem, and it is largely due to his efforts that in recent years so many of our cities have awakened to the importance of a clean and wholesome milk supply. Thus has been born through his efforts the American Association of Medical Milk Commissions, which has, and is still doing great good in supplying our cities with pure clean milk; the organization is of such importance that it is now a national one.

Another important factor in pediatric practice is the personal equation. That so many successful practitioners of general medicine fail in pediatrics, is so obvious as to require no apology for the statement. Diagnostics applied to infanccy and childhood differ materially from that in adults. In infants and young children symptomatology is largely objective, and physical signs must be interpreted, in accordance with the anatomic and physiologic conditions to the age and development. Not only is the pediatrician a medical sociologist, but a medical educator as well, it is this phase of his work which is most difficult. To teach mothers the proper hygiene and care of their children and to strictly adhere to the physician's orders is indeed a strenuous task. In no branch of medicine does success depend so much upon the hearty co-operation of the parents as in pediatrics. A large percentage of mothers can not be educated; we have to do everything for them, care, nursing, the preparation of the food, and the subsequent protection of the child. Cure and prevention go hand in hand. Education as applied to pediatrics and to the reduction of infant mortality is a new necessity. It has become more necessary during the last twenty years because mothers of the last generation were physically more competent and capable than are those of the present day. Our social system is at fault, in that it imposes a greater nervous strain upon parents. It is in this fertile field that a state or county pediatric society would accomplish much good. Let us awaken to the importance of the great international movement which is spreading over the world for the prevention of infant mortality by keeping abreast with the times and organize a county or state pediatric society for the study of these great problems.

If, in setting forth a few of the most important essentials wherein pediatrics differs from the practice of general medicine, the essayist has succeeded in establishing the contention that periatrics is a specialty, the object of this paper has been accomplished.

Fourth National Bank Bldg., Atlanta, Ga.

---

*Discussion of Dr. Rhodes' Paper:—*

Dr. L. B. Clarke said that the profession had long since recognized the fact that children are not little men and women and the colleges have chairs teaching special preparation in care of children. As to the education of the public that special attention should be given to children's diseases he agrees with Rhodes. The case of malnutrition that Rhodes exhibited had evidently been badly handled before carrying it to the specialist. It frequently happens that children are kept until almost dead before proper attention is secured.

The pure milk question has frequently been agitated and we are gradually gaining a pure milk that is better than the pasteurized or sterilized product. Infant mortality due to bad milk has greatly decreased as a result of the activity of the Health Board and several pediatrists.

Dr. W. Z. Holliday called attention to the history of pediatrics. He said the Dr. Abram Jacobi set up the first clinic in the world at Brooklyn, N. Y., in 1860, and the science has grown to its present proportions since then. While in Edinborough four years ago, he was told that Scotland owed what she knew of pediatrics to America.

Dr. George S. Tigner said that the cutting of the teeth came at a time when they are of as much importance to the child as the permanent ones are to the adult. That is the time to get in prevention work against bad teeth and bad digestion. Daily massages should be given to the gums, preventing inflammations there and infantile diseases elsewhere.

Dr. J. W. Duncan, in referring to Dr. Rhodes' case of malnutrition and the peculiar choice it made of foods when allowed a chance to get them, said it showed the force of instinct that existed throughout animal nature. The birds hasten to secure proper food, particularly before a snowstorm, and show another manifestation of this instinct. So far as possible a child's taste should be consulted in feeding it.

Dr. J. S. Derr said the milk supply is of great importance and that restaurants are not careful in handling milk. The cans from which they dip it are open to uncleanliness, as he has personally seen.

Dr. Rhodes closed by saying that while Jacobi had been active in pediatrics 40 years or more, it took many years for enough men to follow his lead to make the science a specialty. Besides the other specialties have been in existence many more years and are generally recognized as such and have been specially taught in the colleges for a long time.

The poorer class of physicians who practice in the slums often know nothing of the special requirements of the child or how to go about getting them if told of their existence.

As to milk supply he is surprised that Atlanta, with all its enterprise, has not certified milk. There are only about 80 Milk Commissions in the United States and Atlanta should have one with plenary authority and responsibility. Thus we could have milk with 10,000 bacilli to the c. c. than the 100,000 to the c. c. which is now the standard. He is satisfied that the present standard is not sufficiently pure for the weak child.

